# New Insights into the Determinants of Specificity in Human Type I Arginase: Generation of a Mutant That Is Only Active with Agmatine as Substrate

**DOI:** 10.3390/ijms23126438

**Published:** 2022-06-09

**Authors:** María-Soledad Orellana, Gonzalo A. Jaña, Maximiliano Figueroa, José Martínez-Oyanedel, Fabiola E. Medina, Estefanía Tarifeño-Saldivia, Marcell Gatica, María Ángeles García-Robles, Nelson Carvajal, Elena Uribe

**Affiliations:** 1 Departamento de Bioquímica y Biología Molecular, Facultad de Ciencias Biológicas, Universidad de Concepción, Casilla 160-C, Concepción 4070386, Chile; morellana@unab.cl (M.-S.O.); maxifigueroa@udec.cl (M.F.); jmartine@udec.cl (J.M.-O.); etarisal@udec.cl (E.T.-S.); marcgatica@udec.cl (M.G.); ncarvaja@udec.cl (N.C.); 2 Facultad de Ciencias de la Vida, Universidad Andres Bello, Santiago 8370251, Chile; 3 Departamento de Ciencias Químicas, Facultad Ciencias Exactas, Universidad Andres Bello, Autopista Concepción-Talcahuano 7100, Concepción 4070386, Chile; gonzalo.jana@unab.cl; 4 Departamento de Química, Facultad de Ciencias, Universidad del Bio-Bio, Concepción 4051381, Chile; famedina@ubiobio.cl; 5 Departamento de Biología Celular, Facultad de Ciencias Biológicas, Universidad de Concepción, Concepción 4070386, Chile; mgarcia@udec.cl

**Keywords:** arginase, arginine, agmatine, determinants of specificity

## Abstract

Arginase catalyzes the hydrolysis of L-arginine into L-ornithine and urea. This enzyme has several analogies with agmatinase, which catalyzes the hydrolysis of agmatine into putrescine and urea. However, this contrasts with the highlighted specificity that each one presents for their respective substrate. A comparison of available crystal structures for arginases reveals an important difference in the extension of two loops located in the entrance of the active site. The first, denominated *loop A* (I129-L140) contains the residues that interact with the alpha carboxyl group or arginine of arginase, and the *loop B* (D181-P184) contains the residues that interact with the alpha amino group of arginine. In this work, to determine the importance of these loops in the specificity of arginase, single, double, and triple arginase mutants in these loops were constructed, as well as chimeras between type I human arginase and *E. coli* agmatinase. In previous studies, the substitution of N130D in arginase (in *loop A*) generated a species capable of hydrolyzing arginine and agmatine. Now, the specificity of arginase is completely altered, generating a chimeric species that is only active with agmatine as a substrate, by substituting I129T, N130Y, and T131A together with the elimination of residues P132, L133, and T134. In addition, Quantum Mechanic/Molecular Mechanic (QM/MM) calculations were carried out to study the accommodation of the substrates in in the active site of this chimera. With these results it is concluded that this loop is decisive to discriminate the type of substrate susceptible to be hydrolyzed by arginase. Evidence was also obtained to define the *loop B* as a structural determinant for substrate affinity. Concretely, the double mutation D181T and V182E generate an enzyme with an essentially unaltered *k_cat_* value, but with a significantly increased *K_m_* value for arginine and a significant decrease in affinity for its product ornithine.

## 1. Introduction

**Figure 1:**
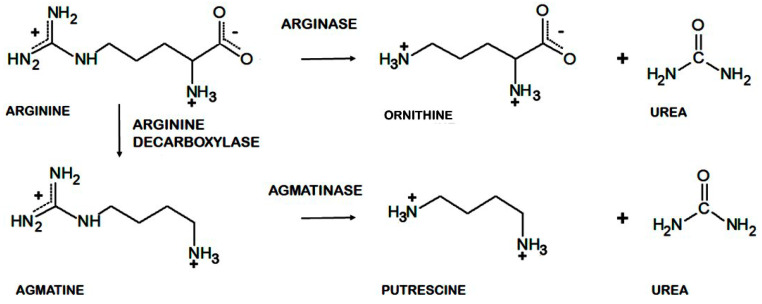
Reactions catalyzed by arginase, agmatinase, and arginine decarboxylase.

Arginase (L-arginine amidinohydrolase, EC 3.5.3.1) catalyzes the hydrolysis of arginine, generating ornithine and urea as products. In a closely related reaction, the agmatinase (agmatine amidinohydrolase, EC 3.5.3.1.1) catalyzes the hydrolysis of agmatine, generating putrescine and urea [1]. Agmatine (1-amino-4-guanidinobutane) is an amine generated by the decarboxylation of arginine, in a reaction catalyzed by the arginine decarboxylase (Figure 1). Despite the similarities between their substrates, both enzymes have a high degree of specificity for the amino acid or the primary amine [2].

Arginase, in addition to its participation in the last reaction of the urea cycle, participates in the regulation of cellular concentrations of arginine and ornithine. The last are essential for the synthesis of nitric oxide, creatine, glutamate, proline, and the polyamines putrescine, spermine, and spermidine. In higher animals, arginase exists in two isoenzymatic forms, which share an approximate 60% similarity in their amino acid sequence, where arginase I is cytosolic and predominant in the liver, while the type II enzyme is mitochondrial and characteristic of extrahepatic tissues, predominantly kidney and prostate [1,2,3,4,5,6,7].

Arginase also plays an important role in the development of lung diseases such as asthma and fibrosis. Indeed, in these pathologies, an increase in arginase activity in the respiratory tract has been observed, decreasing the production of nitric oxide (NO), which has a bronchodilator effect [8,9,10,11]. Consequently, arginase is a potential drug target for diseases in which L-arginine homeostasis and L-arginine-dependent biosynthetic pathways are always disrupted due to the aberrant upregulation of one or both arginase isoenzymes, such as erectile dysfunction, asthma, or atherosclerosis [8,9,10,11,12]. 

Arginases and agmatinases have highly conserved residues in their sequences, which has led to including them within the same protein family [13,14,15,16]. This protein family also includes proclavaminate amidine hydrolase and formimino glutamate hydrolase. Enzymes belonging to this arginase family catalyze hydrolysis reactions with the production of urea or formamide as one of the products. Although it is accepted that these enzymes evolved from a common ancestor and subsequently acquired their substrate specificity [13], so far there are few studies on the molecular basis of such marked differences in specificity [17,18,19,20,21]. In this sense, arginase and agmatinase, despite the close structural analogies between its substrates and the equivalent functions that have been assigned to residues related to catalysis and metal binding, arginine is not a substrate for agmatinase and agmatine is practically not hydrolyzed by arginase. In this regard, it has been observed that rat liver arginase hydrolyzes agmatine with values of *K_m_* 10 times higher than the *K_m_* for arginine and a *k_cat_* approximately 5000 times lower than for arginine [22], and something similar would occur with human arginase type II [23]. Moreover, it has been reported that rat and mouse macrophage arginases do not hydrolyze agmatine [24] and in our laboratory we have not been able to detect the hydrolysis of this compound by human liver arginase I either [17,18].

**Figure 2:**
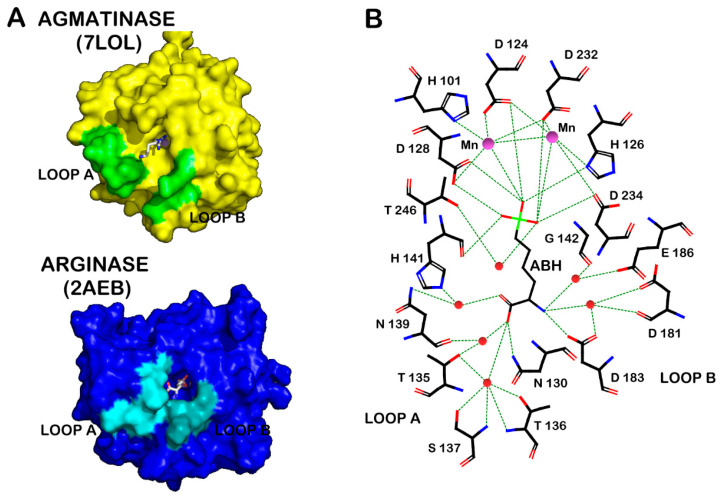
(**A**) Crystal structures of arginase in complex with substrate analogue 2(S)-amino-6-boronohexanoic acid (ABH) complex (PDB code 2AEB) and agmatinase-agmatine complex (PDB code 7LOL). Both complex are showing the *loop A* and *loop B* in different colors. (**B**) Residues involved in the interaction of ABH with arginase and Mn^2+^ center (red spheres represent water molecules).

By superposition of the structures from human liver arginase, *D. radiodurans,* and *E. coli* agmatinase [21,25] (Figure 2A), the overall fold and topology of the enzyme is maintained, in the arrangement and quantity of α-helical and β-sheets structures. Furthermore, the position of the Mn^2+^ and the residues that bind the guanidine group from the substrate are conserved in these structures. However, a clear difference is observed in a loop located at the entrance of the active site, between the residues I129 and L140 (designated as *loop A* in Figure 2A). This loop contains the carboxyl-interacting residues with the substrate arginine [25] (Figure 2B), and it is this part of the substrate that makes the difference between arginine and agmatine. We propose that the differences in this loop would be the key in determining the differences in the specificity for their substrate between arginase and agmatinase. In previous studies by our laboratory, we found that the change of Asn130, present in *loop A* for aspartate, generates a variant of arginase capable of hydrolyzing arginine and agmatine with the same efficiency [17]. Equivalent results were found for arginase type II [18,19], which carried out the mutation of residues Asn130 and Tyr135 in rat arginase and both residues are proposed as determinants of specificity in the enzyme. 

In addition to *loop A*, another loop is also highlighted, which is located on the opposite side of the entrance to the active site (indicated as B in Figure 2A). This *loop B* includes the residues D181-P184, which interact with the alpha amino group of arginine (Figure 2B). In the structure of agmatinase, this loop appears more open than arginase [21]. 

In this work, we performed a detailed analysis of the participation of *loop A* and B in the specificity of human liver arginase for its substrate arginine. To do this, we made single, double, and triple mutants and chimeras of arginase in these loops between human type I arginase and *E. coli* agmatinase. In addition, Quantum Mechanic/Molecular Mechanic (QM/MM) calculations were carried out to study the accommodation of the substrates in in the active site of Chimera A2 mutant enzyme.

## 2. Results

### 2.1. Loop A Mutagenesis in Human Arginase Type I

When analyzing the available crystallographic structures for arginase [5,26,27,28,29,30] and agmatinase [20,21,31], the main differences in their structures lie in two loops located at the entrance to the active site. The ligands for the α-carboxyl group of arginine are located in *loop A*; in this loop the residues N130, S137 and N139 stabilize the alpha carboxyl group of arginine, either by direct hydrogen bonding or with a molecule of water (Figure 2B). Considering that agmatine lacks this carboxyl group, these residues were replaced by site-directed mutagenesis. Specifically, we generated the mutant species N130D, S137C, N139D, and the combinations of double mutants and a triple mutant in which we have simultaneously replaced all three residues. 

**Figure 3:**
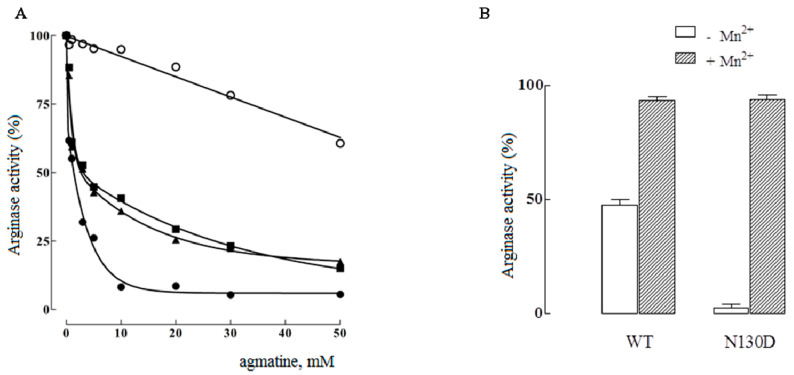
(**A**) Inhibition of arginase activity by agmatine, of the wild species (○) and the mutant species N130D (●), S137C (▲), and N139D (■) of human hepatic arginase. A concentration of arginine of 5 mM was used and the inhibitions were carried out at pH 9.5. The detection of arginase activity was made following the production of ornithine by the method of Chinard [32]. (**B**) Effect of dialysis against EDTA on the catalytic activity of wild-type and mutant N130D species of human liver arginase. The catalytic activity of the dialyzed species was measured at 37 °C for 10 min, in the absence (white bars) and in the presence of Mn^2+^ 2 mM (black bars).

As a result of N130D mutation, the catalytic activity decreased to 17% and the *K_m_* for arginine increased by approximately nine times in comparison to the wild type. This change in catalytic activity and *K_m_* produced a marked decrease in catalytic efficiency, resulting in a *k_cat_*/*K_m_* ratio for arginine 50 times less than for the wild-type enzyme (Table 1). Together with the increase in *K_m_*, the *K_i_* for ornithine increased too in mutant species. In addition, as has also been described, the N130D mutant acquired the ability to hydrolyze agmatine [17,18,19]. This mutant hydrolyzed arginine with practically the same efficiency as agmatine as a substrate, according to the *k_cat_/K*_m_ of 2.48 × 10^3^ M^−1^s^−1^ for arginine and 2.14 × 10^3^ M^−1^s^−1^ for agmatine (Table 1). The point mutations S137C and N139D also generated species with hydrolytic activity on agmatine, but with *k_cat_/K_m_* values of approximately two orders of magnitude lower than those observed for arginine (Table 1), indicating a preference for the substrate arginine in these species. Moreover, the wild-type enzyme was poorly inhibited by agmatine; in contrast, the arginase activity of mutants N130D, S137C, and N139D was markedly sensitive to agmatine inhibition (Figure 3A). The combination of single mutations, to generate double and triple mutations, does not generated a significant variation in the kinetic constants as was observed for the single mutant N130D. As shown in Table 1, in all cases a lower arginase activity was found for all the mutants (10–30% of the activity), with a moderate increase in *K_m_* for arginine and *K_i_* (~7 mM) for competitive inhibition by the ornithine product. On the other hand, all mutant species hydrolyzed agmatine, unlike with the wild-type enzyme, with a catalytic constant between 1 and 3 s^−1^.

Regarding the interaction with the metal cofactor, the mutation N130D generated a species with a lower affinity for Mn^2+^. As shown in Figure 3B, when dialyzing the mutant N130D against EDTA, inactive species either for arginase or agmatinase activities appear in the absence of Mn^2+^. However, this dialyzed mutant is capable of recovering its enzymatic activity in the presence of added metal. This contrasts with the wild-type arginase, which under the same conditions, maintains, in the absence of the metal, approximately 50% of the enzymatic activity. This dialyzed wild type has been reported to contain one Mn^2+^/subunit, according to atomic absorption spectroscopic studies [33]. Therefore, in addition to a change in the specificity of the substrate, the introduction of a negative charge at position 130 alters the interaction of the enzyme with the activator metal, which would explain the lower catalytic activity of the species that contain this mutation.

Since the single, double, or triple mutant species of the residues that appear forming intermolecular interactions with the α-carboxyl group of arginine in the human liver arginase did not generate more profound changes in specificity for the substrate than the observed in the species N130D, we decided to replace all residues contained in *loop A* in a systematic form, by the corresponding residues present in agmatinase from *E. coli*. An electrophoretic and western blot analysis of chimeras A1 to A5 is included in Figure S5 (Supporting Information). The residues involved in these serial mutations were from I129 to P144. Considering that this loop is shorter in the agmatinase, in addition to the indicated mutations, the residues P132, L133, and T134 were eliminated in the arginase. Recombinant chimera proteins were expressed in the *E. coli* strain JM 109 and then purified using the procedures detailed in the Materials and Methods. In Table 2 it is shown, from the analyzed species, that only the chimera A1 (I129T/Nl30Y/Tl31A) possessed arginase activity (3.5% of the enzymatic activity of wild-type arginase), while the chimera A2 (I129T/N130Y/T131A/∆P132-T134) was only active with agmatine as a substrate. The other generated chimeric species in *loop A* do not present catalytic activity neither with arginine nor with agmatine as substrates. In order to rule out that the agmatinase activity shown by the chimeric species A2 corresponded to the endogenous enzyme of the bacterial strain used for expression, the arginase and agmatinase activities were separated by ion exchange chromatography and further corroborated by western blot analysis using an anti-agmatinase antibody from *E. coli*.

In Table 2, it can be seen that the catalytic constant for the chimeric species A1 decreased by approximately 30 times in comparison to the wild-type enzyme, while the *K_m_* for arginine was practically the same. As a consequence, the catalytic efficiency (*k_cat_*/*K_m_*) of the enzyme decreased approximately 50 times. On the other hand, the elimination of residues P132, L133, and Tl34 from the sequence of the chimeric species A1 generated the species A2, with a *K_m_* for agmatine slightly higher than the *K_m_* for arginine of the wild-type enzyme, and an catalytic efficiency three orders of magnitude lower than that exhibited by the wild-type enzyme with arginine as a substrate.

In the chimeric species A2, in addition to the substitution of Asn-130 by Tyr, three residues were eliminated, causing a decrease in the size of this loop, simulating what was observed in agmatinase. Then we analyzed the inhibitory effects of the products and analogues of substrates and products on the catalytic activity of wild-type and chimeric species of arginase. For the chimeric species A1, only active as arginase, the inhibitory effect of the product ornithine and agmatine was analyzed. For the chimeric species A2, only active as agmatinase, the inhibitory effect of putrescine (product of the hydrolysis of agmatine) and its structural analog, ornithine, in addition to arginine as an analog of the substrate, was analyzed. For both species, the effect of guanidine (Gdn^+^), due to its structural analogy with the product urea, was analyzed. Urea was not used directly as an inhibitor, because our activity test is based on the colorimetric determination of this product. Both agmatine and guanidine were found to be competitive inhibitors for the species A1, with a slight but significant decrease in the *K_i_* for agmatine, indicating a slightly higher affinity of the mutant for the structural analog of the substrate. The calculated values were 38 ± 6 mM and 27 ± 2 mM for wild-type and mutant species, respectively. Ornithine turned out to be significantly more inhibitory for the wild-type arginase than for the chimeric mutant A1, competitively inhibiting the chimeric species A1 with a *K_i_* value of 60 ± 2 mM (Table 3), unlike the *K_i_* for the wild-type enzyme that corresponds to 2 ± 0.5 mM. The last result gives us an idea of the magnitude in which the affinity of the A1 chimera for the product decreases, as a consequence of the triple mutation.

**Figure 4:**
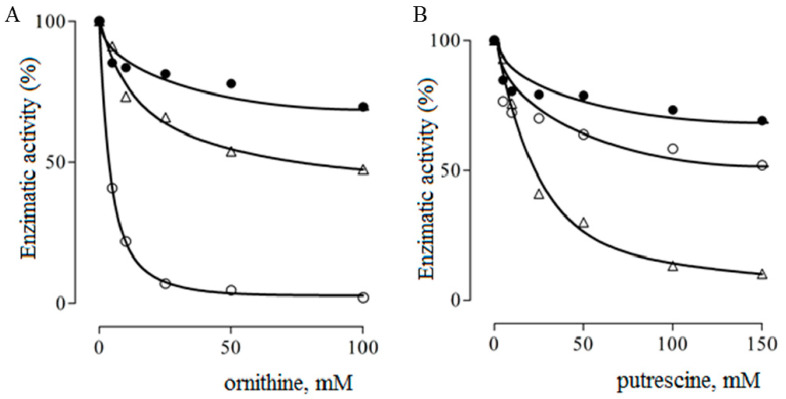
Inhibition of chimeric species A2 (●), wild type arginase (○) and agmatinase of *E. coli* (∆) by ornithine (**A**) and putrescine (**B**), at pH 9.5. The enzymatic activities were measured at 37 °C, with a substrate concentration (arginine or agmatine) of 5 mM and were expressed as a percentage of the the specific catalytic activity in the absence of the inhibitor.

As show in Figure 4A, chimeric species A2 was found to be much more insensitive to inhibition by ornithine than the wild-type arginase. Something similar was observed when analyzing the effect of ornithine on agmatinase from *E. coli* (Figure 4B). However, chimera A2 clearly differs from *E. coli* agmatinase in its sensitivity to putrescine, the hydrolysis product of agmatine. For this chimeric species, a linear competitive inhibition was observed for both arginine and guanidine (Gdn^+^), without observing an important variation in *K_i_* for guanidine inhibition, as shown in Table 3. Regarding putrescine, even though a detailed kinetic study was not carried out, due to the high concentrations necessary to observe significant effects, the partial studies indicated the competitive nature of the inhibition.

### 2.2. Loop B Mutagenesis in Human Arginase Type 1

As already mentioned, another of the major structural differences between arginase and agmatinase lies in a loop located at the entrance of the active site of both enzymes, which we have identified as loop B (Figure 2A). According to crystallographic structures available for arginase [5,26,27,28,29,30] and agmatinase [20,21,31] in the case of human liver arginase, this loop includes four residues; from them two residues stand out of aspartate that would interact with the alpha-amino group of arginine (Figure 2B). 

To analyze the participation of this loop in the specificity of arginase, we have replaced the residues that form *loop B* in arginase, by those corresponding to the equivalent loop in agmatinase from *E. coli*. To do this, first, two point mutations were made (D181T and V182E), then the double mutant D181T/V182E, and finally a Phe residue was inserted after E182. The mutant species were analyzed functionally, using arginine and agmatine as substrates. All species analyzed did not hydrolyze the agmatine. As shown in Table 4, the kinetic properties of arginase were not substantially altered by single mutations at positions 181 or 182. Furthermore, none of the mutations, single or double, produced changes in the catalytic constant (*k_ca_*_t_). However, in the double mutant the *K_m_* for arginine increased 20 times compared to the wild-type enzyme. A significant change was also observed in the interaction of the enzyme with the ornithine product, as shown in Table 4, the double mutant showed mixed-type inhibition, in contrast with the competitive inhibition of the wild-type enzyme. Unlike ornithine, structural analogs lysine and putrescine competitively inhibited the double mutant. On the other hand, the interaction with guanidine, an analog of urea, was not altered, observing a competitive effect and essentially equal *K_i_* values. Although none of the mutants showed activity with agmatine as a substrate, the primary amine is a better inhibitor for the double mutant than for the wild-type enzyme. In addition, the double mutant D181T/V182E plus the Phe residue added after E182, generated a species with a *K_m_* 10 times higher than the wild-type arginase, with a *k_cat_* 24 times lower (Table 4).

### 2.3. QM/MM Calculations to Estimate the Energy Barriers of the Hydrolysis Reaction in Chimera A2

**Figure 5:**
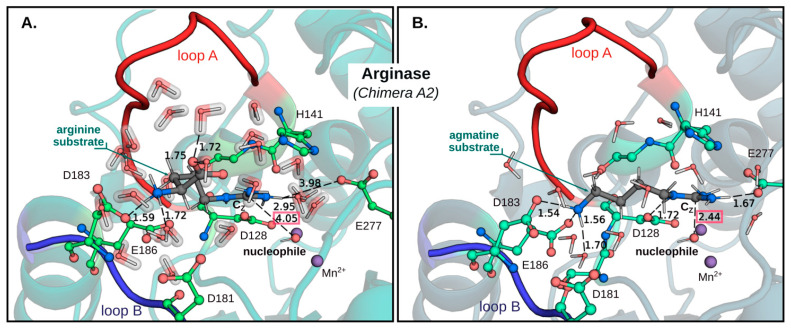
Optimized structure of the Michaelis–Menten complex reactant of Chimera A2 arginase at the B3LYP/6-31+G*:CHARMM36 level. (**A**) Chimera model with arginine substrate. The waters that interact directly with the substrate are highlighted with a shadow representation. (**B**) Chimera with agmatine substrate. The interatomic distances are in Å.

**Figure 6:**
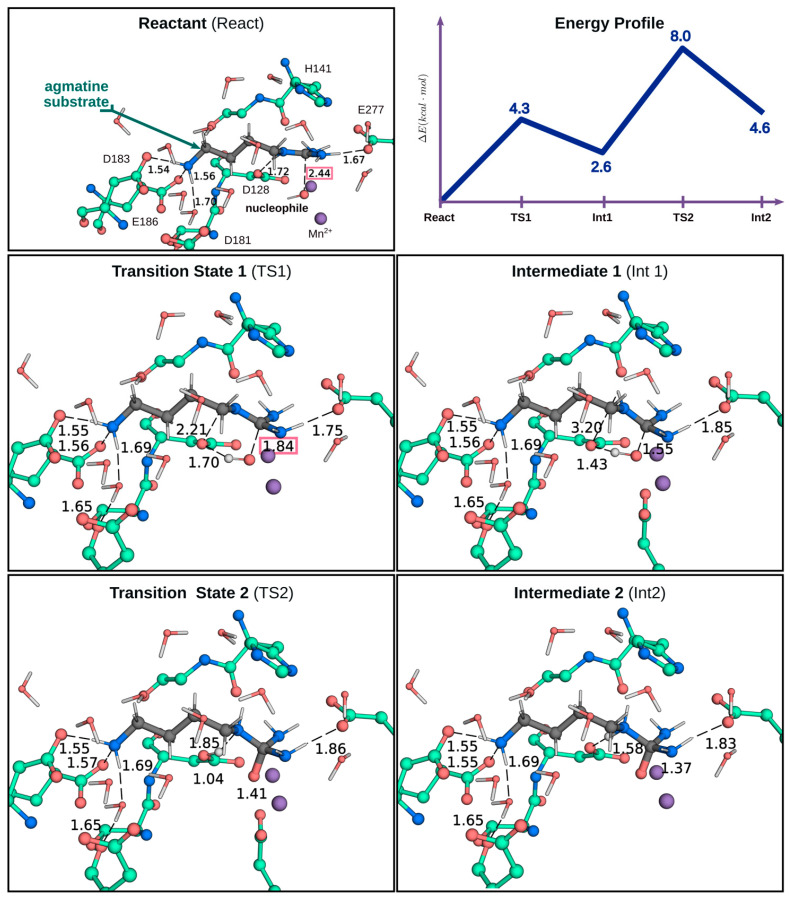
Representative structures and potential energy profile of the reaction path. Schematically describes the hydrolysis of agmatine by the Chimera A2 to the formation of the tetrahedral intermediate (Int2) that forms prior to the release of putrescine.

As previously highlighted, the Chimera A2 was able to hydrolyze agmatine but not arginine. This is not because arginine cannot bind to the enzyme. Therefore, in order to obtain a deeper insight of what catalytic factors could be those that determine this observed difference, SBMD simulation and QM/MM calculations were carried out for the Michaelis–Menten complexes using both agmatine and arginine. The optimized structures of the Michaelis–Menten complexes with agmatine and arginine were obtained through QM/MM calculations, thus allowing a better description of the interactions between the substrates and the residues in the active site. Figure 5 shows the obtained structures of the Chimera A2 with arginine and agmatine. In the complex with arginine (Figure 5A), the terminal amino group of arginine interacts with loop B only through the Asp183 residue. It can also be observed that the α-carboxylic group is exposed to the solvent, losing all the interaction with the enzyme. Moreover, the distance observed between the nucleophile and the CZ carbon of the guanidinium group of arginine from our QM/MM calculations is approximately 4.0 Å, which is not optimal, considering that the distance reported for the complex arginase–arginine in other QM/MM studies is approximately 2.5 Å [34,35]. On the other hand, the agmatine interaction in the active site, as shown in Figure 5B, shows that its amino group interacts with *loop B* through the Asp183 residue and an interaction with Glu186 is also observed. Consequently, the agmatine enters into the active site in a larger degree and this substrate is adequately positioned in the active site, observing a distance of 2.4 Å between the CZ carbon of the guanidinium group of agmatine and the nucleophilic oxygen of the hydroxide anion. Thus, considering that an appropriate catalytic conformation has been obtained to be susceptible to a nucleophilic attack by the hydroxide ion, the minimum energy path (MEP) leading to the formation of the tetrahedral intermediate prior to the cleavage of the CZ-NE bond of agmatine resulting in the formation of putrescine and urea was obtained. Figure 6 shows the representative structures of the critical points along the reaction path and the energy profile of these points. Our results show that the reaction pathway leading to the tetrahedral intermediate that is formed prior to NE-CZ bond cleavage occurs in two stages. The first one, with an energy barrier of approximately 4.3 kcal·mol^−1^ corresponds to the nucleophilic attack of the oxygen of the hydroxide anion on the carbon CZ of the guanidinium group of agmatine, leading to a first tetrahedral intermediate (Int1). Then, in a second stage, the rate-limiting step, proton transfer to the NZ atom of the guanidinium group, occurs with the assistance of the catalytic residue Asp128, to form another tetrahedral intermediate (Int2), as shown in Figure 6. This last stage presents an activation barrier of 8.0 kcal·mol^−1^, which is comparable to the energy barriers obtained for the rate-limiting step in other QM/MM studies carried out on arginine hydrolysis mediated by the arginase enzyme [34,35].

Exploring the nucleophilic attack of the hydroxide ion on the CZ atom of the guanidinium group of arginine in the Chimera A2, starting from the optimized structure shown in Figure 6A, the energy barrier of the reaction increased above +50 kcal·mol^−1^ and no TS was observed (Figure S4), which is consistent with what has been reported in this work regarding the loss of activity towards arginine in the chimera A2.

## 3. Discussion

Previously, we demonstrated that the replacement of the Asn-130 residue by Asp in the *loop A* of human arginase type I, generated a species capable of hydrolyzing, with practically the same efficiency, both arginine and agmatine, in contrast to the wild-type species of the human enzyme that only showed activity with arginine as substrate [17,18]. In this regard, even when it has been reported that rat liver arginase is also active with agmatine, it does it with an efficiency of six orders of magnitude less than for arginine hydrolysis [22]. An equivalent mutation in human arginase type II, species N149D, is only active with agmatine [18]. In principle, the results obtained for the species N130D could be explained considering that the introduction of a negative charge in position 130 of the arginase causes electrostatic repulsion with the α-carboxyl of arginine. However, although this would explain the decrease in the catalytic constant, it does not explain why the agmatine, which lacks the α-carboxyl group, is not hydrolyzed by the wild-type enzyme. Therefore, it is necessary to consider some degree of conformational alteration in the active site, resulting from the mutation introduced at residue 130 of arginase, which would allow the binding of agmatine in a position suitable to be nucleophilically attacked by the hydroxyl group that is permanently attached to Mn^2+^. This would also explain the agmatine inhibition over the arginase activity of the mutant. The postulated conformational change is supported by the differential effects of a dialysis against EDTA, for two hours at 4 °C. Indeed, while this treatment generated native species with a 50% activity in the absence of added Mn^2+^, the mutant species N130D turned out to be inactive and totally dependent on the addition of the activator metal. In this regard, it is necessary to note that, to obtain a totally inactive wild-type species in the absence of the added metal, it requires much more drastic conditions, consisting in a pre-incubation with EDTA 10 mM followed by dialysis for at least 12 h at 4 °C [36]. In summary, although N130 is not a ligand for metal coordination [27], it is evident that the stability of the metal-binding site in this enzyme was affected by the replacement of this residue by aspartate, presumably due to a conformational change generated by the negative charge introduced at position 130 [17,18]. The S137C and N139D mutations, which also compromise residues considered as ligands for the alpha carboxyl group of arginine [26,27], also generated species with a hydrolytic activity on agmatine, although with less catalytic efficiency than the mutant N130D. In addition, the mutants also catalyzed the arginine hydrolysis. The hydrolytic activities over arginine and agmatine, which is presented by the species resulting from double and triple mutations in positions 130, 137, and 139, suggest that the residue at position 130 is very decisive in the specificity of substrate of arginase type one. This is indicated by the relationship between the catalytic efficiencies of the species acting on arginine and agmatine (Table 1). Moreover, Shishova et al. [19] analyzed the effects of replacements of residues that would interact with the alpha carboxyl and alpha amino groups of arginine. According to these authors, as a result of the N130A mutation, the *K_m_* for arginine increased 50-fold and the *k_cat_* was reduced by 37%, while the mutations T135A and T135S did not significantly alter the catalytic efficiency of the enzyme. Even though this residue is also part of the network of hydrogen bonds that stabilize the alpha amine group of the substrate, together with the residues N130, S137, and N139, their interaction with the substrate is mediated by a water molecule, which could explain the low impact that these mutations have on the catalytic efficiency of the enzyme. However, these authors [19], did not analyze the ability of mutant species to interact with agmatine, so it is not possible to establish a more complete comparison with our results.

The studies focused on *loop A* also included the design of chimeric species, resulting from the replacement and elimination of residues close to N130 and the mutation of the same residue. To have some information about the relevance of the negative charge introduced in the case of the mutants described above, N130 was replaced by tyrosine.

The chimeric species Al (I129T/N130Y/T131A) only showed activity with arginine, although with a catalytic efficiency (*k_cat_/K_m_*) approximately 50 times lower than the wild-type enzyme. The lower efficiency is explained, especially, by a lower value of the *k_cat_*, which decreased approximately 30 times in comparison with the wild-type enzyme. Unlike the mutant N130D, there was not a very significant change in the value of *K_m_* for the substrate. Similar results were obtained by Shishova et al. [19]; the mutation N130Y generated a decrease of approximately 20 times in *k_cat_*, with a much more marked increase in *K_m_* for arginine. As shown in Table 2, from all arginase chimeric species generated in this work, only the chimeric species A2 exhibited agmatinase-like activity, in which the mutations I129T, N130Y, and T131A were introduced, and in addition, the residues 132, 133, and 134 were eliminated. The ability to recognize agmatine as a substrate was associated with the removal of these last residues, since the protein that preserves them only presented arginase activity. Considering the obtained results, there is no doubt that the acquisition of the ability to recognize agmatine as a substrate is the result of a conformational change in *loop A*, rather than in the properties of a specific residue. In this regard, it must be considered that the chimera A2 contains a shorter *loop A* than that of the wild-type enzyme. In any case, the inability of the chimeric species to hydrolyze arginine is not due to an inability of this substrate to bind to the enzyme, as this compound behaved as a competitive inhibitor for agmatine hydrolysis, and with a *K_i_* that is in the order of the *K_m_* values determined for the species examined in this work. The inability of the chimeric enzyme to hydrolyze arginine could be explained by an inappropriate positioning to be the target of the nucleophilic attack by the hydroxyl group attached to the activator metal. In fact, the analysis performed by QM/MM calculations showed that the arginine as a substrate did not favor the reaction in the direction of the product formation. Thus, the results of the MEP confirm that an appropriate conformation of the guanidinium group, through its interaction with Glu277, facilitates the nucleophilic attack of the hydroxyl ion, since the energy barrier obtained for this step with agmatine was only 4.3 kcal·mol^−1^.

As we have already mentioned, another of the areas that appears with some degree of variation in the structures of arginase and agmatinase corresponds to *loop B*. According to the available crystallographic structures, highlighted in this area are the residues D181 and D183, which interact with the α-amino group of arginine. These interactions are established by means of a direct hydrogen bond with D183 and hydrogen bonds mediated by a water molecule with both aspartate residues (Figure 2B) [30]. When these residues were exchanged for those present in the *E. coli* agmatinase, the greatest kinetic change was produced by the replacement of D181T and V82E. In this species, the *k_cat_* value did not change, but the catalytic efficiency (*k_cat_/K_m_*) decreased significantly as a result of a 20-fold increase in the *K_m_* value for arginine. A similar result was reported by Shishova et al. [19], when performing the D183N mutant in human arginase, they observed a 27-fold increase in Km with a reduction of only 12% in *k_cat_*. 

**Figure 7:**
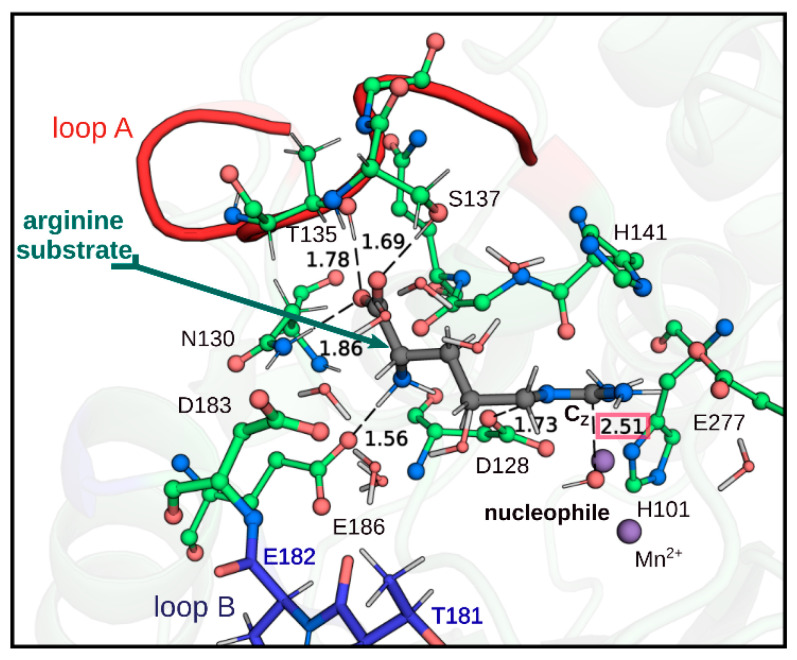
Optimized structure of the Michaelis–Menten complex reactant with arginine substrate of arginase D181T/V182E double mutant at the B3LYP/6-31+G*:CHARMM36 level. The distances are in Å.

None of the point mutations of these residues generated changes as significant as those observed when carrying out the joint mutation of residues 181 and 182. The substitution of both residues generates a local alteration slightly greater than the point mutations, caused by the loss of the D181 interaction and the negative charge added in the position of a hydrophobic residue, generating a very significant increase in *K_m_*. Molecular modeling studies using the ModLoop server [37], showed that simple modifications do not alter the location of the main chain, with only small changes in the disposal of the residues. However, when we analyzed the D181T/V182E double mutation, the main chain changes its orientation with respect to the wild-type species, leaving the residues 181 and 183 at a distance that prevents the formation of the network of interactions with the amine group of the substrate [30]. These studies could explain the increase in *K_m_* value for arginine binding. However, maintaining the *k_cat_* value suggests that the correct positioning of the substrate in the active site of the enzyme was not substantially altered. Actually, as it is shown in Figure 7, our QM/MM calculations on the Michaelis–Menten complex with arginine in the double mutant enzyme show that the α-carboxylic group of arginine keeps the interaction with the A-loop through the residues Asp130, Thr135, and Ser137. In addition, the amino group interacts with the protein only through the hydrogen bond with the residue Glu186. Although no interactions with the B-loop are observed, the substrate is correctly positioned in the active site cavity, interacting correctly with Glu277 through the guanidinium group and with a distance of 2.5 Å between the CZ atom of the guanidinium group of arginine and the nucleophilic oxygen of the hydroxide anion. The latter gives atomistic–molecular support to what is observed experimentally, since, although the double mutation affects the binding mode of arginine, it can still enter into the active site adequately, keeping an appropriate distance to be susceptible to nucleophilic attack by the hydroxide anion.

By inserting a phenylalanine residue after position 182, the main chain of this loop would move further away from the amino group of the substrate, losing another point of interaction. Wild-type arginase has a hydrogen bond between the oxygen atom of the carboxyl group at position 183 and one of the hydrogen atoms of the substrate amino group. With this modification, the interactions with the alpha carboxyl group and the bimetallic center would probably suffer some alteration, which could alter the correct positioning of the substrate for efficient hydrolysis, a fact that would explain the marked decrease in *k_cat_* in this mutant.

Together with the change in *K_m_* for the double mutant D181T/V182E, a very significant variation was observed in the type of inhibition by the ornithine product. While the product behaves as a competitive linear inhibitor for the wild type, the inhibition of the double mutant turned out to be of a mixed type. It is possible that in the mixed inhibition generated by ornithine, it could act as a product and also as a dead-end inhibitor by binding to the enzyme–arginine complex. In any case, a predominantly competitive character is clearly observed in the inhibition by ornithine (*K_is_* < *K_ii_*), which indicates that the amino acid binds with a much higher affinity to the active site of the double mutant than to a different position. As a product, the ornithine binds with a considerably higher affinity to the wild-type enzyme, as indicated by the *K_is_* value and is approximately 75 times higher for the double mutant. The *K_i_* value for lysine inhibition (42 mM) also increased significantly with respect to the 5 mM value calculated for the wild-type species.

When evaluating the interaction with guanidine, as a urea analog, it was found that all the mutant species generated were competitively inhibited, not observing significant variations in the respective *K_i_* values. The crystallographic structures available for arginase indicate that arginine binds with its guanidino group oriented towards manganese ions at the bottom of a cavity ~15Å and the alpha carbon is located in the periphery of the active site [28]. The effect produced by the double mutation would be restricted to the area of the periphery of the active site. If the interaction with the hydroxyl ion bound to the metal or the affinity for the guanidino group of the substrate had been affected, the *k_cat_* or *K_i_* values for guanidine inhibition would have been affected too, which was not observed experimentally.

In conclusion, the results obtained in this work allow us to propose that *loop A* constitutes a structural determinant for substrate specificity in arginase. We have altered the specificity of arginase, generating a chimeric species that is only active with agmatine as a substrate. In parallel, we have found a critical region for the affinity of the binding of arginase with arginine, which corresponds to *loop B*, where the residues that interact with the alpha amino group of arginine are found. These residues would constitute the structural determinant for the affinity of the enzyme–substrate interaction. 

## 4. Material and Methods

### 4.1. Materials

All reagents were from Merck Chemical Co, Darmstadt, Germany. Restriction enzymes, as well as enzymes and reagents for the polymerase chain reaction (PCR) were obtained from Promega, Madison, Wisconsin, USA. The plasmid pBluescript II KS(+), containing the human liver arginase type I cDNA was kindly provided by Dr. Stephen Cederbaum (University of California, Los Angeles, CA, USA).

### 4.2. Enzyme Preparations

Bacterial cultures were grown with shaking at 37 °C in Luria Broth media, in the presence of ampicillin (100 μg/mL). The wild-type and mutant human liver arginase cDNAs were directionally cloned into the pBluescript II KS(+) expression vector and then expressed in *E. coli* JM109, following induction with 1 mM isopropyl β-D-thiogalactopyranoside. The bacterial cells were disrupted by sonication on ice (5 × 30 s pulses), centrifuged for 20 min at 12,000× *g*, and the obtained supernatant was precipitated with ammonium sulfate (60% saturation). The pellet, collected by centrifugation at 12,000× *g* for 10 min, was re-suspended in buffer 5 mM Tris–HCl (pH 7.5) containing 2 mM MnCl_2_ and dialyzed for 6 h at 4 °C against the same buffer. After incubation with 5 mM MnCl_2_ for 10 min at 60 °C, the enzyme variants were purified as previously described [17]. The purity of the enzymes was evaluated by SDS–PAGE stained by Coomassie blue and immunodetection using an anti- rat arginase I antibody.

### 4.3. Site-Directed Mutagenesis

The single, double, triple, and chimera variants of human arginase type I were obtained by PCR, using the QuickChange site-directed mutagenesis kit (Stratagene, La Jolla, CA, USA); the plasmid pBluescript II KS(+) containing the human liver arginase cDNA was used as template. To obtain the chimeric species of human arginase type I, serial mutations were made in each loop to be modified, using as template DNA the wild-type arginase gene or the previous done mutation, subcloned in the plasmid pBluescript II KS (+). Specifically, the residues present in the arginase structure were replaced by those corresponding to the agmatinase sequence of *E. coli*.

### 4.4. Enzymatic Assays and Kinetic Studies

Enzyme activities were determined by measuring the production of urea from L-arginine in 50 mM glycine–NaOH (pH 9.5) and 2 mM Mn^2+^. All assays were initiated by adding the enzyme to a previously equilibrated buffer with the substrate in solution (the concentrations of used substrates were between 0.2–3 fold the *K_m_*). Urea was determined by a colorimetric method with α-isonitrosopropiophenone [38]. The protein concentrations were determined by the Bradford’s method, with bovine serum albumin as standard. Initial velocity and enzymatic inhibition experiments were performed in duplicate and repeated three times. The inhibitory patterns were initially determined by double reciprocal plots and re-plots of intercepts and slopes versus inhibitor concentrations. Data were then fitted to the appropriate equations for competitive or mixed-type inhibitions, by using nonlinear regression in GraphPad Prism version 5.0 for Windows (GraphPad Software Inc., San Diego, CA, USA). Velocities are expressed as μmol of urea/10 min.

### 4.5. Molecular Modeling and QM/MM Calculation of Arginase Enzyme with Mutation in A-Loop

All the mutants were structurally modeled by comparative modeling, using the Modeller 9.20 (Andrej Sali, San Francisco, CA, USA) software, and the structure of the human arginase type I as template (PDB ID: 2AEB). The protocol included a slow refinement using molecular dynamics simulations into the Modeller script. The resulting models were analyzed by Pisa and visually inspected using PyMol. All the images were created using PyMol. 

The protonation states at pH 7.0 of the ionizable residues were determined by PROPKA3.1 program [39]. Then, the system was solvated with a sphere of pre-equilibrated water with a radius of 25 Å centered on the CZ atom of guanidinium group of the substrate (See Figure S1 in the Supporting Information). All crystallographic waters beyond the sphere were removed along with any water molecule in which its oxygen wass closer than 2.5 Å to any heavy atom of the protein. Stochastic boundary molecular dynamics (SBMD) simulations [40] were carried out for the systems with a total simulation time of 100 ns at 300 K using an integration step of 1 fs.

In SBMD simulations the system was divided in three regions: The dynamic region contains all the atoms laying in a radius of 20 Å from the CZ atom of the guanidinium group of the substrate. The Langevin region has been defined with a radius of 25 Å, i.e., contains all the atoms surrounding the dynamic region from 20 to 25 Å. The rest of the system corresponds to the reservoir. Prior to SBMD simulations, the system was relaxed by 300 steps of energy minimization using adopted basis Newton–Raphson algorithm (ABNR). The sizes of the buffer and dynamics zones are large enough to ensure that the active site and all the possible rearrangement in its surroundings during the reaction were correctly modeled. All simulations were carried out with CHARMM package [41] by using the CHARMM36 all-atom force field [42].

During the SBMD simulation, no significant displacement of the amino acids located in the active site were observed (see Figures S2 and S3 in the Supporting Information). Based on this fact, the final structure of the simulation was taken as a single representative configuration for the QM/MM calculations. The system was partitioned into a Quantum Mechanics parts (QM) that includes the arginine or agmatine substrates, the residues Asp128, His141, Glu277, and the hydroxide ion (nucleophile) that is coordinated with two Mn^2+^ ions. The rest of the system was treated at the molecular mechanical level (MM) (Figure S4 in the Supporting Information). The Mn^2+^ ions were excluded from the QM zone, since it has been shown that their role is purely structural and no relevant changes have been observed in their electronic density during the catalysis [34]. Three link atoms were used, each one of them located along the C_α_-C_β_ bond of Asp128, His141, and Glu277 residues. The geometry of the QM subsystem was described at B3LYP/6-31+G* level of theory and CHARMM36 all-atom force field was used for the MM treatment. CHARMM and Q-CHEM packages [43] were employed to carry out the calculations and an electronic embedding scheme was used for the QM/MM treatment [44]. For the optimization of the geometry of the Michaelis complexes with agmatine or arginine, an active region of 25 Angtroms radius centered on the CZ atom of the guanidinium group was defined. Every residue within this region could move freely, while everything outside this zone was fixed.

The Minimum Energy Path (MEP) of the reaction catalyzed for the A2 Chimera with agmatine was obtained using the Conjugate Peak Refinement (CPR) algorithm [45] as implemented in the TREK module of the CHARMM package. The CPR algorithm reconstructs the entire path connecting the reactant and product endpoints. Only the energy function and its gradient are required, guaranteeing that the energy barrier is not affected by a biased description of the process and, most importantly, ensuring that energy maximum along this adiabatic path is a true saddle point.

## Figures and Tables

**Table 1:** Kinetic properties of the single, double, and triple mutants of the residues that bind the α-carboxyl of arginine in human arginase type I.

	Arginine				Agmatine		
Enzyme	*k_cat_*	*K_m_*	*k_cat_/K_m_*	*K_i_^orn^*	*k_cat_*	*K_m_*	*k_cat_/K_m_*
	(s^−1^)	(mM)	(M^−1^s^−1^)	(mM)	(s^−1^)	(mM)	(M^−1^s^−1^)
N130D	33	13.3	2.5 × 10^3^	7.8	3.0	1.4	2.1 × 10^3^
S137C	75	3.2	2.3 × 10^4^	7.6	1.8	7.5	2.4 × 10^2^
N139D	64	4.4	1.4 × 10^4^	6.2	1.3	5.3	2.5 × 10^2^
N130D-S137C	23	6.5	3.5 × 10^3^	7.1	2.3	1.7	1.3 × 10^3^
N130D-N139D	33	6.1	5.4 × 10^3^	6.4	1.6	1.1	1.4 × 10^3^
S137C-N139D	83	10.8	7.7 × 10^3^	8.4	1.1	0.9	1.2 × 10^3^
N130D-S137C-N139D	4	6.9	5.8 × 10^2^	5.4	0.9	1.6	5.6 × 10^2^
WT-arginase	190	1.5	1.3 × 10^5^	1.0	n/a		
*E. coli*-agmatinase	n/a				120	1.1	1.1 × 10^5^

The values of the kinetic parameters indicated in this table correspond to the results of two experiments performed in duplicate and the standard deviations were not greater than 5%. n/a: indicates no activity with the respective substrate.

**Table 2:** Kinetic properties of the wild-type human arginase type I and chimeric species of *loop A*.

	Arginine			Agmatine		
	*K_m_* (mM)	*k_cat_* (s^−1^)	*k_cat_/K_m_* (M^−1^s^−1^)	*K_m_* (mM)	*k_cat_* (s^−1^)	*k_cat_/K_m_* (M^−1^s^−1^)
WT-arginase	1.5 ± 0.5	190 ± 10	1.3 × 10^5^		n/a	
*E. coli* agmatinase		n/a		1.1 ± 0.2	120 ± 10	1.1 × 10^5^
Chimera A1 I129T/N130Y/T131A	2.5 ± 0.5	6.2 ± 0.4	2.4 × 10^3^		n/a	
Chimera A2 I129T/N130Y/T131A+∆ P132-T134		n/a		6 ± 1	1.1 ± 0.2	1.8 × 10^2^
Chimera A3 I129T/N130Y/T131A ∆ P132–T134/N139F/L140D		n/a			n/a	
Chimera A4 I129T/N130Y/T131A/∆ P132–T134/T135N/T136G/S137C/G138E/N139F/L140D		n/a			n/a	
Chimera A5 (residues I129 t P144 according to *Loop A* in agmatinase)		n/a			n/a	

The values of the kinetic parameters indicated in this table correspond to the results of two experiments performed in duplicate and the standard deviations were not greater than 5%. n/a: indicates no activity with the respective substrate.

**Table 3:** Inhibition studies of the chimeric species A1 and A2 of human arginase type I.

	Substrate	Inhibitor	Inhibition Type	*K_is_* (mM)
WT-arginase	Arginine	Guanidine	Competitive	56 ± 4
Chimera A1	Arginine	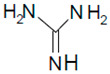	Competitive	38 ± 6
Chimera A2	Agmatine		Competitive	40 ± 6
WT-arginase	Arginine	Agmatine	Competitive	42 ± 5
Chimera A1	Arginine	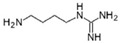	Competitive	27 ± 2
WT-arginase	Arginine	Ornithine	Competitive	2 ± 0.5
Chimera A1	Arginine	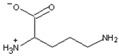	Competitive	60 ± 2
Chimera A2	Agmatine	Arginine	Competitive	9 ± 3
		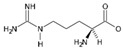		

The values of the kinetic parameters indicated in this table correspond to the results of two experiments performed in duplicate and the standard deviations were not greater than 5%. Chimeric species: A1 (I129T/N130Y/131A mutations); A2 (I129T/N130Y/T131A-∆P132-T134).

**Table 4:** Kinetic characterization and inhibition studies of mutant species of *loop B* of type I human arginase.

		Arginine		Agmatine	Ornithine Inhibition	*K_is_* (mM)	Guanidine Inhibition	*K_is_* (mM)
	*K_m_* (mM)	*k_cat_* (s^−1^)	*k_cat_/K_m_* (M^−1^ s^−1^)			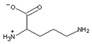		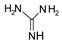
WT-arginase	1.5 ± 0.5	190 ± 10	1.26 × 10^5^	n/a	Competitive	2 ± 0.5	Competitive	60 ± 2
D181T	5.3 ± 0.8	191 ± 8	3.6 × 10^4^	n/a	Competitive	1.6 ± 0.3	Competitive	85 ± 10
V182E	2.4 ± 0.2	187 ± 10	7.7 × 10^4^	n/a	Competitive	3.2 ± 0.2	Competitive	89 ± 12
D181T/V182E	32 ± 5	188 ± 10	5.8 × 10^3^	n/a	Mixed	*K_is_* = 71 ± 12 *K_ii_* = 203 ± 28	Competitive	80 ± 5
D181T/V182E + ins F	20 ± 5	8.1 ± 1	3.5 × 10^2^	n/a	Competitive	18 ± 6	Competitive	70 ± 10

The values of the kinetic parameters indicated in this table correspond to the results of two experiments performed in duplicate and the standard deviations were not greater than 5%. n/a: indicates no activity with the respective substrate.

## Data Availability

Not applicable.

## Supplementary Materials

The following supporting information can be downloaded at: https://www.mdpi.com/article/10.3390/ijms23126438/s1.
