# Supplementary material for: New Insights into the Determinants of Specificity in Human Type I Arginase: Generation of a Mutant That Is Only Active with Agmatine as Substrate
Source: Int J Mol Sci. 2022 Jun 9;23(12):6438. doi: 10.3390/ijms23126438 (PMC9224512; doi:10.3390/ijms23126438)
Supplement: Supplementary file 1 [file ijms-23-06438-s001.zip › ijms-1726172-supplementary.pdf]

SUPPORTING INFORMATION

New Insights into the Determinants of Specificity in Human Type I Arginase: Generation of a Mutant That Is Only Active with Agmatine as Substrate

María-Soledad Orellana[1,2], Gonzalo A. Jaña[3], Maximiliano Figueroa[1], José Martínez-Oyanedel[1], Fabiola E. Medina[4], Estefanía Tarifeño-Saldivia[1], Marcell Gatica[1], María Ángeles García-Robles[5], Nelson Carvajal[1] and Elena Uribe[1].*

1. Departamento de Bioquímica y Biología Molecular, Facultad de Ciencias Biológicas, Universidad de Concepción, Casilla 160-C, Concepción 4070386, Chile.; morellana@unab.cl (M.-S.O.); maxifigueroa@udec.cl (M.F.); jmartine@udec.cl (J.M.-O.); etarisal@udec.cl (E.T.-S.); marcgatica@udec.cl (M.G.); (N.C.) ncarvaja@udec.cl
2. Facultad de Ciencias de la Vida, Universidad Andres Bello, Santiago 8370251, Chile
3. Departamento de Ciencias Químicas, Facultad Ciencias Exactas, Universidad Andres Bello, Autopista Concepción-Talcahuano 7100, Concepción, Chile; gonzalo.jana@unab.cl
4. Departamento de Química, Facultad de Ciencias, Universidad del Bio-Bio, Concepción 4051381, Chile; famedina@ubiobio.cl
5. Departamento de Biología Celular, Facultad de Ciencias Biológicas, Universidad de Concepción, Concepción 4070386, Chile; mgarcia@udec.cl

*Correspondence: Elena Uribe, 56-41-2204428, e-mail: auribe@udec.cl.

Method Section

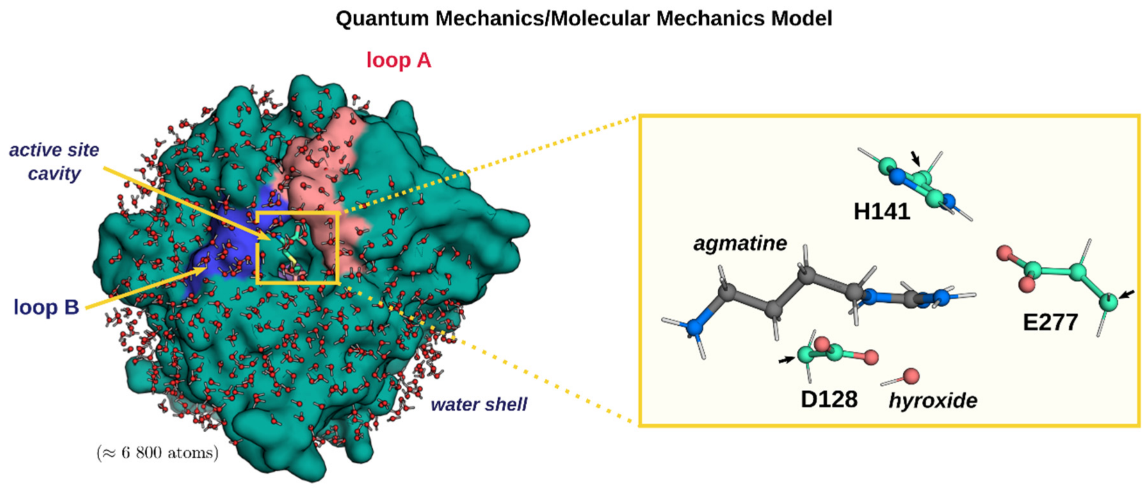

Figure S1. Arginase A2 chimera system complexed with agmatine substrate. The water shell around the enzyme is represented with ball-and-sticks and the QM region is shown by a close-up.

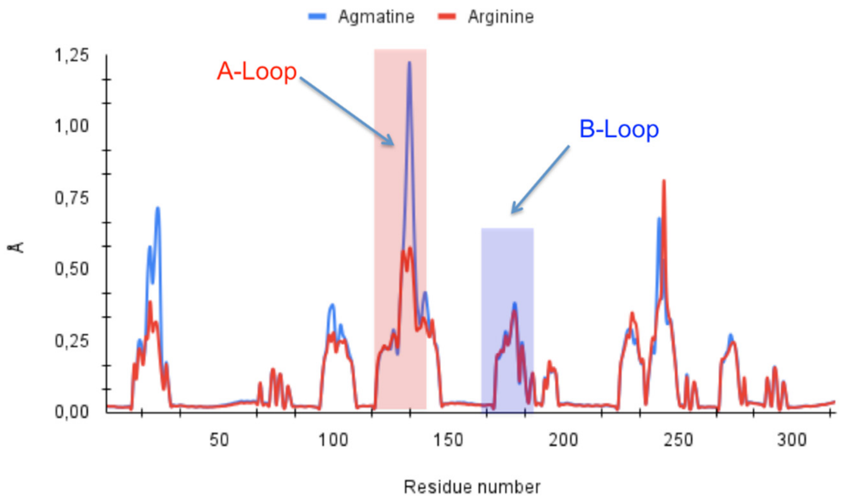

Figure S2. Root mean square fluctuation per residue obtained from SBMD simulation of A2 Chimera in complex with Agmatine(blue line) and Arginine (red line). Residues showing zero RMSF are located in the reservoir zone.

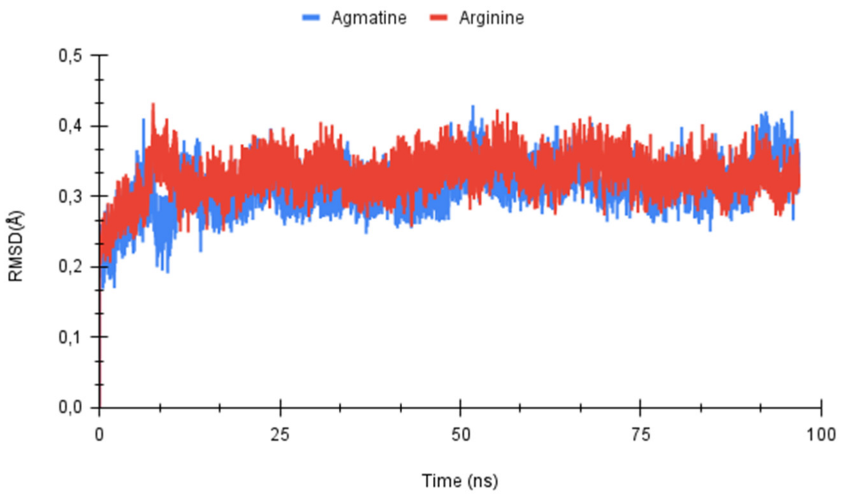

Figure S3. Root mean square desviation obtained from SBMD simulation of A2 Chimera in complex with Agmatine(blue line) and Arginine(red line).

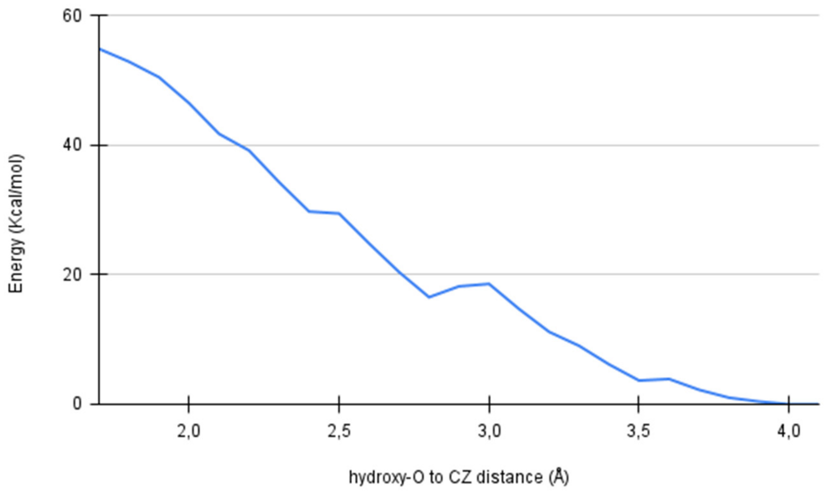

Figure S4. Energy scan for the nucleophilic addition of the hydroxyde-oxygen to CZ atom of guanidinum group of the arginine in the Chimera A2.The energy of the linear transit scan were calculated at the B3LYP/6-31+G*:CHARMM36 level.

Results Section

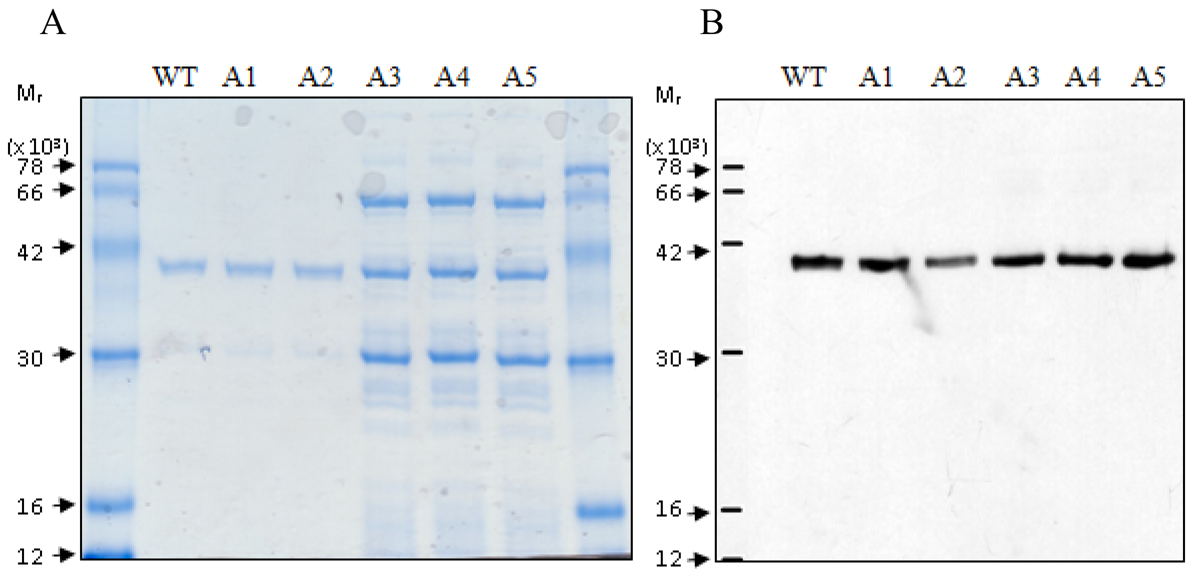

Figure S5. Analysis of expression of chimeric species of loop A of arginase. **A.** Electrophoretic analysis under denaturing conditions (12% SDS-PAGE) of wild type arginase species (WT) and chimeric species of loop A 1 to 5 (A1 to A5). **B.** Western blot analysis with an anti-human liver arginase antibody, transferred from a polyacrylamide-SDS gel of the same conditions described in (A).
